# Dental Students’ and Dental School Graduates’ Practical Skills: An International Survey of Perceptions of National Dental Associations in Europe

**DOI:** 10.3290/j.ohpd.b4997035

**Published:** 2024-02-20

**Authors:** Thomas Gerhard Wolf, Simona Dianišková, Edoardo Cavallé, Rena Aliyeva, Maria-Grazia Cagetti, Guglielmo Campus, James Deschner, Norina Forna, Duygu Ilhan, Marco Mazevet, Anna Lella, Paulo Melo, Paula Perlea, Angela Rovera, Anton Sculean, Nikolai Sharkov, Ariel Slutsky, António Roma Torres, Mare Saag

**Affiliations:** a Professor, Department of Restorative, Preventive and Pediatric Dentistry, School of Dental Medicine, University of Bern, Bern, Switzerland (T.G.W.); Department of Periodontology and Operative Dentistry, University Medical Center of the Johannes Gutenberg-University Mainz, Germany.; b Professor, ERO-FDI president, Department of Orthodontics, Medical Faculty, Slovak Medical University, Bratislava (S.D.).; c ERO-FDI President-elect, board member, National Association of Italian Dentists (ANDI), Roma, Italy.; d Board Member, Azerbaijan Stomatological Association, Baku, Azerbaijan (R.A.).; e Professor, Department of Biomedical, Surgical and Dental Sciences, University of Milan, Milan, Italy (M.G.C.).; f Professor, Department of Surgery, Microsurgery and Medicine Sciences, School of Dentistry, University of Sassari, Sassari, Italy (G.C.).; g Professor and Head, Department of Periodontology and Operative Dentistry, University Medical Center of the Johannes Gutenberg-University Mainz, Germany (J.D.).; h Professor, Department Implantology, Removable Restorations, Dental Medical Faculty, Grigore T. Popa University of Medicine and Pharmacy, Iasi, Romania (N.F.).; i Assistant Professor, FDI Councillor, Department of Periodontology, Istanbul Medipol University, Turkish Dental Association, Turkey (D.I.).; j Chief Executive Officer, Les Chirurgiens-Dentistes de France, Paris, France (M.M.).; k FDI Councillor, Polish Dental Society, Warsaw, Poland (A.L.).; l Professor, FDI Councillor, Faculty of Dental Medicine, EpiUnit, Institute of Public Health, University of Porto, Porto, Portugal (P.M.).; m Professor and Head, Department of Endodontics, Carol Davila University of Medicine and Pharmacy, Bucharest, Romania (P.P.).; n Dental Physical Sciences Unit, Centre for Oral Bioengineering, Institute of Dentistry, Queen Mary University of London, London, UK; National Association of Italian Dentists (ANDI), Rome, Italy (A.R.).; o Professor and Head, Department of Periodontology, School of Dental Medicine, University of Bern, Bern, Switzerland (T.S.).; p Assistant Professor, FDI President-elect, Bulgarian Dental Association, Sofia, Bulgaria (N.S.).; q Board member, The Israeli Dental Association, Tel Aviv, Israel (A.S.).; r Board member, Portuguese Dental Association, Porto, Portugal (A.R.T.).; s Professor, Chairman, Institute of Dentistry, Faculty of Medicine, University of Tartu, Tartu, Estonia (M.S.).

**Keywords:** dental association, graduate, international, practical skills, student

## Abstract

**Purpose:**

Dental students learn knowledge and practical skills to provide oral health care to the population. Practical skills must be maintained or continuously developed throughout a professional career. This cross-sectional survey aimed to evaluate the perception of practical skills of dental students and dental-school graduates by national dental associations (NDAs) in international comparison in the European Regional Organization of the FDI World Dental Federation (ERO-FDI) zone.

**Materials and Methods:**

A questionnaire of 14 items collected information on pre-/postgraduate areas.

**Results:**

A total of 25 countries participated (response rate: 69.4%), with 80.0% having minimum requirements for practical skills acquisition and 64.0% starting practical training in the 3rd year of study. In countries where clinical practical work on patients begins in the 2nd year of study, practical skills of graduates are perceived as average, starting in the 3rd year of study as mainly good, starting in the 4th as varying widely from poor to very good. In total, 76.0% of respondents feel that improvements are needed before entering dental practice. Improvements could be reached by treating more patients in dental school (32.0%), increasing the quantity of clinical training (20.0%), or having more clinical instructors (12.0%). In 56.0% of the countries, it is possible to open one’s own dental practice immediately after graduation, and in 16.0%, prior vocational training is mandatory.

**Conclusions:**

All participating countries in the ERO-FDI zone reported practical training in dental school, most starting in the 3rd year of study. The perception of practical skills of dental students and dental-school graduates among NDAs is very heterogeneous. Reasons for the perceived deficiencies should be further explored.

The goal of every university dental school is to teach knowledge and practical skills in all relevant topics to create professionals able to promote and improve the oral health of the population.^8,16^ Although dentists must continually develop and maintain their skills throughout their professional life, the objective of dental education is also to provide safe and independent, i.e., without the influence of third parties, dental care to patients.^12^ In Europe, the focus on problem-based learning in teaching is increasing.^7,18^ Therefore, essential elements such as competency-based education and its concepts have recently come to the fore.^4,7,23^ A shift from a discipline-oriented/instructor-centered approach to competence-based education with a focus on learning outcomes and authentic assessment has been met with great interest and widespread support from both university educators and policymakers in the health professions.^4,13,18,23,26^

However, the question of what constitutes an optimal basic dental education is still not conclusively settled, although there is a clear influence from constant technical advancements. In addition to a high degree of judgment, emotional challenges,^2^ and technical skills,^5^ clinical outcomes as well as complex material procedures play a very large role.^27^ Compared to students in other healthcare fields, dental students perform invasive and irreversible procedures during their undergraduate courses.^6^ Nevertheless, competency can also be defined according to the needs of dental practice. Instead of focusing only on university teaching to acquire skills and values, knowledge of practice management is also necessary, as it is the only way to establish and operate an independent dental practice.^20,22^

According to the national dental associations (NDAs) in numerous countries, practice owners who hire dentists immediately after graduation describe deficits in practical skills, which underlines the demand on a professional policy that dental education must be improved.^1,17^ NDAs, along with dental schools, play an important role in finding solutions to global oral-health challenges.^32^ Nationally as well as internationally, disparities are large due to a lack of standardised dental curricula or inadequate objective assessment systems. Postgraduate education is intended to fill knowledge gaps and equip dentists with necessary skills in the early years of their careers.

The aim of the present cross-sectional survey was therefore to investigate how NDAs perceive the practical skills of their dental students immediately after graduation and to identify whether there are any known problems or challenges that prevent them from starting an independent dental practice.

## Materials and Methods

### General Information and Participating Countries

The WHO Europe Region is one of a total of six WHO areas worldwide, and includes a total of 53 countries stretching from the Atlantic to the Pacific,^29^ with a population of over 922 million people.^30^ Compared to the European Union with 27 member states, the WHO Europe Region is more than twice as large in terms of population, with approximately 445 million people.^9^ Of these 53 countries, a total of 36 countries are currently represented in the European Regional Organisation of the FDI World Dental Federation (ERO-FDI),^10^ with a population of approximately 466 million. The ERO-FDI represents approximately 500,000 dentists,^28^ which is nearly half of all dentists represented in the FDI World Dental Federation.^11^ The ERO-FDI is one of a total of five FDI sub-organisations: African Regional Organisation (ARO), Asia-Pacific Regional Organisation (APRO), European Regional Organisation (ERO), Latin American Regional Organisation (LARO), and North American Regional Organisation (NARO).

### Questionnaire

The study was conducted using a questionnaire in English that was sent to the contact persons of the NDAs. The questionnaire items are listed in the results section. Members of the ERO-FDI working group “Relation between dental practitioners and universities” and researchers invited to the working group prepared and tested the questionnaire in the working-group meetings before it was sent out. The questionnaire contained brief information about the purpose of the survey and was accessible via a form that was e-mailed to participants. The questionnaire was sent out in February 2022 with several reminders to the officers of the respective NDAs of the ERO-FDI member states. After several reminders, data collection was completed on July 31, 2022. All procedures conformed to the ethical standards of the local research commission and the 1964 Declaration of Helsinki and its subsequent amendments, or comparable ethical standards. The questionnaire was developed following similar questionnaires in the literature.^14,28,32,33^ Questions were asked about the start of clinical training in dental school, the presence of minimum requirements, perceptions of practical skills, perceptions of dental-school graduates by the NDAs, opportunities to improve practical skills, permission to work after graduation, need for professional training/internship, specialty training programs after graduation, and organisations offering professional training/internship courses. Other items concerned post-graduation aspects: final examination of practical skills, receipt of a salary for work, tuition fees, and organisations offering dental internship/professional training courses for a fee. The questionnaire was pre-tested on a small sample of 10 dental teachers/lecturers and members of the working group to evaluate comprehensibility. After completing the questionnaire, they were contacted to find out if they had experienced any difficulty in understanding the questions and were given a comprehension score from 1 (extreme difficulty) to 5 (no difficulty), with a result of 4.4 ± 0.5. Suggestions were proposed by participants in this pilot test about the wording of the items, and the questionnaire was modified accordingly.

### Data Analysis

Questionnaire responses were entered into Excel (Microsoft; Redmond, WA, USA) 2019 for Mac. Data were cleaned and then transferred to STATA16 (StataCorp LLC; College Station, TX, USA) for statistical analysis. Absolute and relative frequencies were calculated for each item. Differences in proportion were evaluated using the *χ*^2^ test or Fisher’s exact test if a cell had a value of less than five. Although some cells have a value of zero, the Fisher’s exact test was calculated. Multiple tests were calculated for post-hoc estimates such as the number of observed frequencies, expected frequencies, percentage, and contribution to the *χ*^2^. The significance level was set at p < 0.05.

## Results

In total, 25 NDAs from the 36 member countries of the ERO-FDI region participated in this study. The following countries participated: Austria, Azerbaijan, Belgium, Bulgaria, Czechia, Cyprus, Estonia, Georgia, Greece, Germany, Hungary, Israel, Italy, Kyrgyzstan, Latvia, the Netherlands, North Macedonia, Poland, Portugal, Romania, Slovakia, Slovenia, Switzerland, Turkey, the United Kingdom and Northern Ireland.

**Fig 1:**
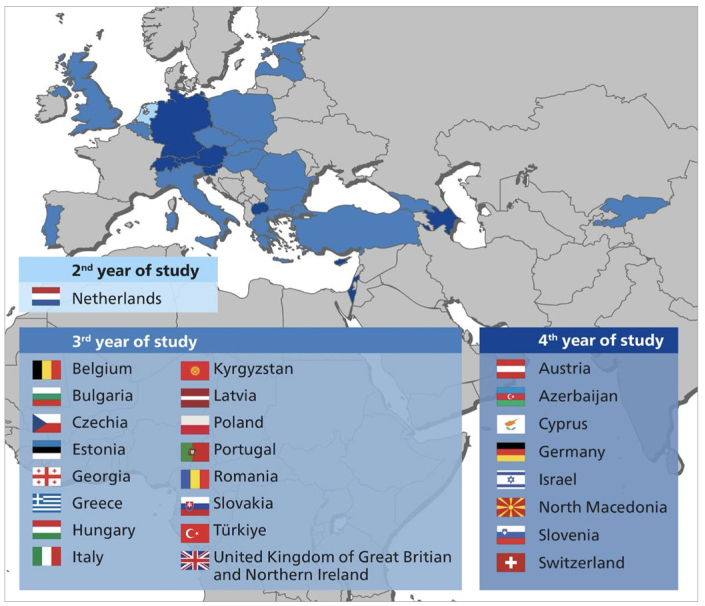
Participating countries of the ERO-FDI zone with information on the start of clinical practical skills training (on patients) by year of study (2nd, 3rd or 4th).

A map of the European geographic region with participating countries is shown in Fig 1. This map also includes information on the academic year in which students in the corresponding country start clinical practical work in their studies (i.e., having patients under supervision), with most countries starting training in practical skills in the 3rd academic year (Fig 1).

Information on the start of clinical training at dental school, the presence of minimum requirements, perceptions of practical skills of dental-school graduates by NDAs, and questions about opportunities to improve practical skills are presented in Table 1. Due to missing data on different questions, the total number of respondents per question varies. Only a few countries allow students to be employed in dental practices and treat patients already during their studies (16.7%). There are minimum requirements for the acquisition of practical skills in most countries (83.3%). Practical skills of dental-school graduates starting clinical training in the second year are perceived as average (4.2%), mostly good when starting clinical work in third year (66.7%), and vary widely from poor to very good when starting clinical work in the fourth year (25.0% each). 76.0% of NDAs think that dental graduates should improve their skills before entering dental practice. NDAs think that the number of patients treated (32.0%), the number of clinical instructors (12.0%), the number of hours of clinical training (20.0%) or by treating in dental practices (8.0%) can be improved (Table 1).

**Table 1:** Information about start of clinical training at dental school, existence of minimum requirements, perception about practical skills, National Dental Associations’ (NDA) perception of dental graduates’ practical skills, and questions about possibilities to improve practical skills

Year of study Number (percentage)	2nd n (%)	3rd n (%)	4th n (%)
Are students allowed to be employed by dental offices and treat patients during their undergraduate university enrolment?			
No	0 (0.00)	12 (80.00)	7 (87.50)
Yes	1 (100.00)	2 (13.33)	1 (12.50)
No answer	0 (0.00)	1 (6.67)	0 (0.00)
Total	1 (100.00)	15 (100.00)	8 (100.00)
Fisher’s exact test = 0.286			
Are there minimum requirements for obtaining practical skills at your universities?			
No	0 (0.00)	2 (13.33)	0 (0.00)
Yes	1 (100.00)	12 (80.00)	7 (87.50)
No answer	0 (0.00)	1 (6.67)	1 (12.50)
Total	1 (100.00)	15 (100.00)	8 (100.00)
Fisher’s exact test = 0.816			
What is your average perception about the practical skills of dental graduates in your country?			
Very bad	0 (0.00)	0 (0.00)	0 (0.00)
Bad	0 (0.00)	1 (6.67)	2 (25.00)
Average	1 (100.00)	3 (20.00)	2 (25.00)
Good	0 (0.00)	10 (66.67)	2 (25.00)
Very good	0 (0.00)	1 (6.67)	2 (25.00)
Total	1 (100.00)	15 (100.00)	8 (100.00)
Fisher’s exact test = 0.175			
Does your NDA feel that the dental graduates should improve their skills before starting to practice?			
Yes	1 (100.00)	11 (73.33)	7 (87.50)
No	0 (0.00)	4 (26.67)	1 (12.50)
Total	1 (100.00)	15 (100.00)	8 (100.00)
Fisher’s exact test = 0.700			
Do you consider that practical skills of dental graduates could be improved by increasing … ?			
number of patients in the university clinic	0 (0.00)	5 (55.56)	3 (42.86)
number of clinical teachers	0 (0.00)	2 (22.22)	1 (14.29)
number of hours of practical training in the curriculum	1 (100.00)	1 (11.11)	3 (42.86)
practice in outpatient clinics	0 (0.00)	1 (11.11)	0 (0.00)
Total	1 (100.00)	9 (100.00)	7 (100.00)
Fisher’s exact test = 0.536			

Permission to work after graduation, professional development/internship requirements, postgraduate specialty training programs, and organisations offering professional development/internships can be found in Table 2. Dental graduates are allowed to start their own practice in 56.0% of countries and must continue to work under the supervision of dentists in the dental practice where they are employed in 12.0% of the countries. Of the respondents, 20.0% see a need for vocational training/internship after graduation, in 16.0% of the participating countries a vocational training is mandatory. Entry into a specialist training program directly after graduation is possible in 60.0% of the countries; in 36.0%, a vocational training/internship or working as a general dental practitioner is needed. Organisations offering a fee-based training program exist in 24.0% of the countries, and no organisations exist in 64.0%.

**Table 2:** Information about permission to work after graduation, need for vocational training/internship, specialist training program after graduation and organisations offering vocational training/internship courses

In your country, after graduation the dentist can: n (%)	
start a self-reliant dental practice	14 (56.0%)
should work under supervision	3 (12.0%)
should go for vocational training/ internship	5 (20.0%)
No answer/not available	3 (12.0%)
Total	25 (100.0%)
Do you see the need for the introduction of vocational training/ internship after dental graduation? n (%)	
Yes, mandatory	4 (16.0%)
Yes, on a voluntary basis	7 (28.0%)
No	3 (12.0%)
No answer/not available	11 (44.0%)
Total	25 (100.0%)
Can the new graduates enter residency (specialist training program) immediately after graduation?	
Yes	15 (60.0%)
No, only after vocational training/internship	2 (8.0%)
No, only after practicing as a general practitioner for one year	3 (12.0%)
No, only after practicing as a general practitioner for two years	4 (16.0%)
No answer/not available	1 (4.0%)
Total	25 (100.0%)
Are there organisations offering vocational training/internship courses for a fee? n (%)	
Yes	6 (24.0%)
No	16 (64.0%)
No answer/not available	3 (12.0%)
Total	25 (100%)

Table 3 refers to post-graduation information: final practical skills examination, receipt of a paycheck, tuition, and organisations offering paid dental internships/professional training. In 80.0% of the countries, a final practical skills examination exists, in 20.0% of the participating countries it does not. In 60.0%, dentists receive a salary for their work after graduation, while in 44.0% of the countries, additional tuition is needed after graduation. In 24.0% of the countries, there are organaistions that offer vocational training or dental internship.

**Table 3:** Information about after graduation: final examination of practical skills, receiving salary for work, tuition fees, and organisations offering dental internship/vocational training courses for a fee

Is there a final examination of practical skills on graduation? n (%)	
Yes	20 (80.00)
No	5 (20.00)
No answer /Not available	0 (0.00)
Total	25 (100.00)
Do they receive a salary for their work after graduation? n (%)	
Yes	15 (60.00)
No	8 (32.00)
No answer /Not available	2 (8.00)
Total	25 (100.00)
Do they pay any tuition fees for working after graduation? n (%)	
Yes	11 (44.00)
No	13 (52.00)
No answer /Not available	1 (4.00)
Total	25 (100.00)
Are there any organisations offering dental internship/vocational training courses for a fee after graduation? n (%)	
Yes	6 (24.00)
No	16 (64.00)
No answer /Not available	3 (12.00)
Total	25 (100.00)

## Discussion

The purpose of this cross-sectional study was to collect information about the perceptions of NDAs regarding the practical skills of their national dental students during dental school and after graduation. Problems and challenges that prevent the establishment of an independent dental practice were to be identified.

### Main Findings

Most participating countries begin clinical practical-skills training in the 3rd year of study, only one country in the 2nd year, and the remaining countries in the 4th year. In most countries, minimum requirements exist for the acquisition of practical skills. The NDAs regard these practical skills as average when clinical practical work starts in the second year of study, predominantly good when starting in the third year of study, and from poor to very good when starting in the fourth year of study. Most NDAs also believe that dental-school graduates should improve their skills before entering dental practice, e.g., by treating more patients, increasing the number of clinical instructors or hours of clinical training, or time spent treating patients in dental practices.

Confirming these findings, final year students were most confident performing simpler procedures and practices in which they had the most clinical experience^17^ and less confident in more complex procedures and practices in which they had the least clinical experience.^17^ This is also consistent with the evidence of perceived inability of newly graduated dentists to make decisions, raising questions about the state of learning outcomes.^1^ Thus, increasing the clinical experience with complex procedures could help boost students’ confidence in these areas during their final year of study.^17^ Dental graduates face a new situation, work atmosphere, and environment, which brings numerous challenges in the transition from dental school to professional life.^24^ The discrepancy between university and professional practice may lead to uncertainty and stress among recent graduates,^16^ so that actual practical skills may not be displayed.^21,24^ Soft skills such as communication can also have a significant impact on dental practice, which is why numerous curricula in the last decade have increasingly included training in such psychological skills.^3,25^ Trust in the dentist, satisfaction with treatment and thus compliance can be improved through communication.^3^ Also, the various challenges that a dental practice brings with it, such as practice management, billing or general economic as well as ecological aspects suddenly play a completely different and very important role in the dental practice. Of course, the SARS-Co-2/COVID-19 pandemic and its worldwide impact, especially in 2020 and 2021, should not go unmentioned. The existence or installation of online learning platforms together with communication technologies enabled dental schools to maintain at least theoretical teaching through various periods of national lockdowns and restrictions on teaching.^19^ Although these online learning opportunities are a useful complement to teaching for knowledge transfer, it should not be forgotten that national restrictions have meant that practical training had to be reduced or even completely suspended in many places. Although recent dental graduates have excellent theoretical knowledge and have mastered basic clinical skills, these young dentists still lack experience with complex treatments, which is why they are reluctant to start their own dental practice immediately after graduation.^15,19^ The concerns expressed by some stakeholders regarding clinical skills and possible inadequate preparation for self-employment^15,19^ can be confirmed in this study but cannot be substantiated in terms of their background. Clinical competence is seen as a fundamental aspect of preparedness for practice, which is composed of various elements such as health, mental health, and emotional support.^15^ Even though graduates are accepted as “safe beginners”, expectations as well as perceptions of what constitutes preparedness for practice differ considerably.^15^ There is also evidence that instructors tend to have higher expectations of graduates than the standards required in learning outcomes reflect; thus, the lack of preparation relates primarily to complex cases where experience is lacking.^15,19^

### Limitations

Several limitations must be pointed out. Only two-thirds of the countries in the ERO-FDI zone took part in the survey, which means that the ERO-FDI zone is not reflected in a representative way. Despite this limitation, the overall sample size of the international study is adequate with a total of 25 participating countries. The fact that the questionnaire was presented to the contact persons only in English is excluded as a limitation, since the contact persons or their secretariats of the participating countries should be proficient in English, as this is the main language of communication in the ERO-FDI and FDI World Dental Federation, although simultaneous translation into other languages, i.e., German and French, is also usually provided in the general meetings of ERO-FDI. A certain bias due to self-selection or desirability or response tendencies of the contact persons are possible and cannot be excluded. In addition, it must also be mentioned that the drastic impact of the SARS-CoV-2/COVID-19 pandemic was not investigated by this study but should play a role in interpretation of the findings due to a possible bias in perception of the NDAs. Therefore, a repetition of this study in a stabilised, non-pandemic situation seems reasonable in a few years. Despite several meetings of the working group, misunderstandings or misinterpretations may have occurred, although the questionnaire was previously validated by the working group. Also, the issue of overly complex or insufficiently deep questions for quantitative presentation without qualitative investigation should be mentioned as a limitation in this cross-sectional study. Another advantage of this quantitative data collection, besides the ability to intervene quickly and cost efficiency, is that it represents attitudes or opinions, which are given by perception of practical skills in this study.

### Outlook

More than a decade ago, dental schools were expected to provide greater support to NDAs and to collaborate on undergraduate curriculum development, dental workforce issues, and negotiation of professional matters with regulatory agencies.^31^ However, collaboration between universities and NDAs was evaluated as needing improvement.^31^ Although the profile, core competencies and skills of a European dentist have already been defined and are expected to be followed by European dental schools, competencies may vary from university to university.^7^ Especially against the background of constant technological novelties and further development of the dental profession, the involvement of European dental schools, NDAs, professional dental associations, and societies as well as ministries of health is extremely useful to contribute to and support not only the harmonisation of the curriculum in Europe but also the goal of improving the quality of studies.^7,15^ Due to lack of evidence in other areas of the FDI World Dental Federation, no global statements can be made about NDAs’ perceptions of dental students’ practical skills; therefore, further analysis is needed. Although the results cannot be unrestrictedly generalised, the data provide an overview of NDAs’ perceptions of their students’ practical skills and offer NDAs, universities and policymakers a deeper look at the issue and considerations for possible solutions.

## Conclusions

Within the limitations of the international cross-sectional questionnaire study in the ERO-FDI zone, the following conclusions can be drawn:

- All 25 participating countries in the ERO-FDI zone have practical training in dental school, with most starting in the 3rd year of study;
- Perception of practical skills of dental students and graduates among NDAs is very heterogeneous;
- Practical skills of dental-school graduates are perceived by NDAs as average when clinical practical work starts in the 2nd year of study, good when it starts in the 3rd year, and varying between poor to very good when it starts in 4th year of study;
- The reasons for the perceived deficits should be further explored, thus further research is needed.
